# *Ex vivo* comparative investigation of suprachiasmatic nucleus excitotoxic resiliency

**DOI:** 10.12688/f1000research.125332.2

**Published:** 2023-01-09

**Authors:** Debalina Acharyya, Joanna Cooper, Rebecca A. Prosser

**Affiliations:** 1 Biochemistry & Cellular and Molecular Biology, University of Tennessee Knoxville, Knoxville, TN, 37996, USA; 2 The Center for Vascular and Inflammatory Disease, University of Maryland School of Medicine, Baltimore, MD, 21201, USA

**Keywords:** suprachiasmatic, excitotoxicity, NMDA, cortex, brain slice

## Abstract

**Background:** Glutamate signaling in the brain is regulated by release, reuptake, and receptor responsiveness. In diseased conditions, glutamate signaling can exceed normal regulatory processes, giving rise to a condition called excitotoxicity. Although regional differences in the excitotoxic effects of glutamate in the brain have been reported, the extent and characteristics of these potential differences are not clear. Here we compared the excitotoxic resiliency of the suprachiasmatic nucleus (SCN), anterior hypothalamus (AH) and cortex.

**Methods:** We treated acute brain slices containing either the SCN and AH or the cortex from adult male mice at different times across the diurnal cycle with varying concentrations of N-methyl-D-aspartate (NMDA), NMDA+ α-amino-3-hydroxy-5-methyl-4-isoxazolepropionic acid (AMPA) or control medium. The extent of cell damage was assessed using propidium iodide (PI), a cell death marker.

**Results:** The results indicate that all three brain regions exhibited increasing cell damage/death when treated with increasing concentrations of NMDA. However, higher concentrations of NMDA were needed to significantly increase cell damage in the SCN compared to the cortex and AH. All three brain regions also exhibited greater cell death/damage when treated in the nighttime compared to the daytime, although the SCN exhibited increased cell death during a more restricted time interval compared to the AH and cortex.

**Conclusions:** Together, these data confirm previous studies showing excitotoxic resiliency in the SCN, while extending them in two ways. First, we demonstrate a dose-dependency in excitotoxic susceptibility that differentiates the SCN from the surrounding AH and the cortex using a brain slice preparation. Second, we demonstrate a diurnal rhythm in excitotoxic susceptibility with a broadly similar phase across all three brain regions. These data increase our understanding of the extent and nature of the SCN excitotoxic resiliency, which will inform future studies on the cellular mechanisms underlying this phenomenon.

## Introduction

Glutamate is the primary excitatory neurotransmitter in the brain and as such is critical for proper neural functioning. ^1^ Under physiological conditions, the optimal extracellular glutamate concentration is maintained by a balance between astrocytic reuptake and neural/astrocytic release. ^2^ However, certain disease conditions, including ischemic stroke, traumatic brain injury and neurodegenerative diseases, are accompanied by an excess accumulation of extracellular glutamate. For example, an increase in extracellular glutamate is seen after severe trauma that persists for more than four days. ^3^ This leads to over-activating N-methyl-D-aspartate (NMDA) and α-amino-3-hydroxy-5-methyl-4-isoxazolepropionic acid (AMPA) receptors and ultimately to cell damage and death, a phenomenon known as excitotoxicity. ^4^

Although excitotoxicity is initiated by glutamate, Ca ^2+^ is the primary mediator of excitotoxic damage. Excessive glutamate-induced influx of Ca ^2+^ disrupts normal metabolic processes, creates oxidative stress, and activates cell death pathways. ^5^ Importantly, the degree of cell death induced by excess glutamate/Ca ^2+^ signaling may differ across the brain. The characteristics and mechanism(s) underlying these regional differences are not clearly understood, but determining intrinsic/extrinsic mechanisms that enhance excitotoxic resiliency could help in developing treatments for this debilitating pathology.

The suprachiasmatic nucleus (SCN), site of the primary circadian oscillator in mammals, is thought to be resilient to excitotoxic damage. This was first reported in 1980 by Peterson *et al.*, ^6^ when kainic acid injections into the lateral hypothalamus and ventrolateral geniculate led to widespread death while sparing the SCN. Subsequent studies demonstrated that immortalized rat SCN cells (SCN 2.2 cells) exhibit resiliency to glutamate-induced excitotoxic damage compared to hypothalamic (GT1-7) cells. ^7^^,^^8^ Although informative, these latter studies investigated dispersed cells in culture, which lack the normal *in situ* cellular and extracellular environment. Thus, whether the same resiliency differences and potential underlying mechanism(s) occur under intact tissue conditions is unclear. Moreover, the resiliency of the SCN has not been quantitatively compared to other brain regions, such as the cortex, known to exhibit high excitotoxic susceptibility. ^8^ Lastly, although the SCN exhibits circadian rhythms in many cellular mechanisms, its excitotoxic resiliency has not been investigated across different times of the day. Therefore, we investigated excitotoxic resiliency using acute murine brain slices, comparing the effects across multiple brain regions, different times of the day and using different strengths of excitotoxic stimuli. Our results show 1) the SCN is more resilient to excitotoxic damage compared to the adjacent anterior hypothalamus (AH) and the cortex; 2) the resiliency of all three brain regions is dose-dependent; and 3) the SCN, AH and cortex all exhibit a diurnal rhythm in excitotoxicity resiliency.

## Methods

### Brain slice preparation

Acute coronal brain slices (500 μm) containing both the SCN and adjacent AH or the cortex were prepared from adult male C57Bl/6 mice (Envigo; Indianapolis, IN) housed under a 12:12 light-dark cycle with food and water available *ad libitum.* Although specific cortical areas were not targeted for this study, all cortex slices were from the midline and most contained somatosensory cortex immediately posterior to bregma. All procedures with the animals were approved by the University of Tennessee IACUC committee, protocol #1453, and all efforts were made to minimize suffering of the animals. Slices were prepared between Zeitgeber Time 0-2 (ZT 0-2, where ZT 0=lights on and ZT 12=lights off in the donor animal colony) or ZT 10-12 and placed in Hatton-style brain slice dishes perfused with Earle’s Balanced Salt Solution (EBSS) (MP Biomedicals; OH, USA) supplemented with glucose and sodium bicarbonate, pH 7.4, gassed with 95% O _2_, 5% CO _2_ and maintained at 37°C as described elsewhere. ^9^ Depending on the experiment, brain slices were maintained for approximately 4 or 10 h prior to experimental treatment.

### Excitotoxic treatment

At the designated time relative to the animal colony light cycle (ZT 6, 12, 16 or 22), perfusion of the brain slice chamber was paused, and the slices were left untreated or treated by bath application of EBSS supplemented with NMDA (50 μM – 10 mM) (Sigma Aldrich; MO, USA) for 1 h. In some experiments, slices were treated with EBSS supplemented with 500 μM NMDA + 50 μM AMPA (Sigma Aldrich). Following the 1 h treatment, the perfusion medium was replaced with normal EBSS, and perfusion was resumed for an additional 3 h.

### Propidium iodide staining and tissue fixation

At the end of the 3 h, brain slice perfusion was switched to EBSS containing 4.6 μg/ml propidium iodide (PI) (Invitrogen; OR, USA) for an additional 2 h. At the end of this period, the tissues were placed in 4% paraformaldehyde (Electron Microscopy Sciences; PA, USA) for 10 min, then transferred to a 30% sucrose solution and incubated overnight at 4°C. The following day, the slices were embedded in optimum temperature cutting compound (Tissue Tek; CA, USA), and the resulting tissue blocks were stored in -80°C until sectioning.

### Tissue sectioning and imaging

The tissues were sectioned (10 μm) with a cryostat and the sections were collected on gelatin-coated slides and stored in -80 ^ο^C. For the imaging, slides containing the tissue sections were air-dried and then washed in phosphate buffered saline. After washing, the slides were cover- slipped with Vectashield mounting medium (Thermofisher Scientific; CA, USA) containing 4′,6′-diamidino-2-phenylindole (DAPI) and the edges were sealed with nail-polish. Fluorescence images were acquired using a Leica DM6000B microscope at 10× magnification. Similar settings were maintained for the acquisition of all images.

### Image analysis

All image analyses were performed using ImageJ software (NIH). Given the lack of consensus in the literature for evaluating the extent of cell death/damage in histological images, we compared the utility of three different image analysis protocols: intensity-based cell count (IBCC), particle analysis cell count (PACC) and mean gray value (MGV). For all three protocols, the images acquired were first split into individual channels: blue (DAPI, 405 nm) and red (PI, ~570-610 nM). Using the blue channel, the region of interest (ROI) was marked in each section. The subsequent steps for each procedure are detailed below. In all cases, one image was analyzed per brain slice for each experimental condition and a total of three to eight replicates were analyzed for each experimental condition (replicate numbers varied across experiments). Images were analyzed by at least three individuals blind to the treatment, with greater-than-85% consistency across all individuals. The mean of the individual analyses for each image was used for statistical analysis.

*Intensity-based cell count (IBCC)*

The total number of cells in each ROI were manually counted using the blue channel. The red channel exhibited a range of PI intensities, consistent with cells having varying degrees of excitotoxic its induced cell damage and death. Based on a random sampling of these intensities across the entire image, thresholds were determined that separated the cells into three categories: Grade 1 (little or no PI staining; healthy cells), Grade 2 (moderate PI staining; damaged cells) and Grade 3 (intense and sharply delineated PI staining; dead cells). ^10^ The percentage of cells in each category was determined by dividing the number of cells in each category by total number of cells in the ROI.

*Particle analysis cell count (PACC)*

The background was subtracted from both the red and the blue channels. Next, the threshold was adjusted in the blue channel to delineate individual cells, using the watershed option on ImageJ to separate clumped cells. The total number of cells in the ROI were counted in the blue channel using the particle analysis option. Using the red channel, the threshold was adjusted twice in ImageJ, once to show only Grade 3 cells and a second time so that Grade 2+3 cells were shown. In both cases, the particle analysis option on ImageJ was used to count the resulting shown cells in the ROI. The number of Grade 3 cells was subtracted to determine the number of Grade 2 cells. The number of Grade 1 cells was calculated by subtracting the number of Grade 2+3 cells from the total number of cells. The percentage of cells in each category was calculated by dividing the number of cells in each category by the total number of cells in the ROI.

*Mean gray value (MGV)*

The background was subtracted from both the red and the blue channels. Next, the mean gray value in the ROI was determined for each channel. The PI:DAPI ratio was calculated to determine the relative amount of PI staining across the entire ROI without reference to different degrees of staining intensity. The percentage of Grade 2+3 (unhealthy) cells and Grade 1 (healthy) cells were calculated by the formulas:

$$\text{Grade}\;2+3\;\text{cells}\;\left(\text{unhealthy cells}\right)=100\times \left[\mathrm{PI}/\text{DAPI}\right]$$

$$\text{Grade}\;1\;\text{cells}\;\left(\text{healthy cells}\right)=100\times \left[1-\mathrm{PI}/\text{DAPI}\right]$$

### Graphing and statistical analysis

The data were graphed, and statistical analysis was performed using GraphPad Prism software (Version 8.0.1). Spearman Pearson correlation was performed to compare the three-imaging analysis (IBCC, PACC and MGV) procedures. Note that the experiments were not designed to test for differences in the percentage of cells across Grades ( *i.e.*, whether the percentages of Grade 1, 2 and 3 cells differ from each other). Instead, the experiments were designed to test whether there were significant changes within a particular cell Grade based on experimental condition. Therefore, one-way ANOVA (testing for changes in cell percentages within Grades under different experimental conditions) followed by Tukey test was performed to investigate inter-region differences under control conditions and to compare dose responses to increasing NMDA concentrations within each brain area. Based on our results demonstrating statistical differences across regions under control conditions, subsequent analyses were restricted to within-region comparisons. One-tailed Student’s T-tests (only assessing treatment-induced decreases in healthy cells or increases in damaged/dead cells; Microsoft Excel) were used, as indicated, testing for changes within Grades, within each region of interest, across the various experimental conditions. Differences were considered statistically significant at *p*<0.05.

## Results

### PI imaging protocols identify regional differences in cell health under control conditions

**Figure 1.**
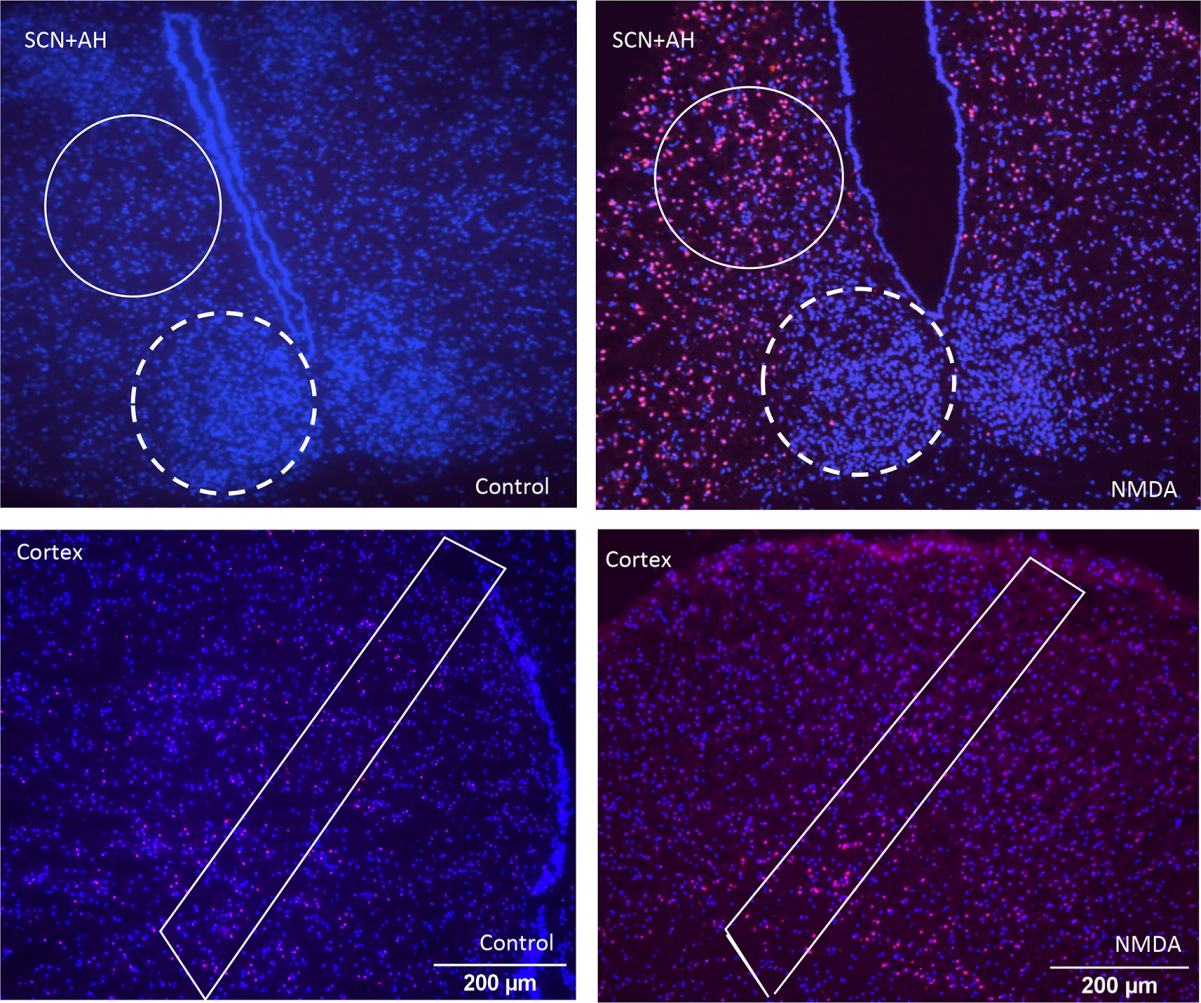
Representative images of propidium iodide (PI) and DAPI staining of different regions of the brain under control and NMDA conditions. Top row: SCN (dashed line) and anterior hypothalamus (AH) (solid line) under control and NMDA conditions respectively. Bottom row: cortical sections under control and NMDA conditions respectively. Differences in the intensity of PI staining are evident across the different images based on region and treatment condition.

**Figure 2.**
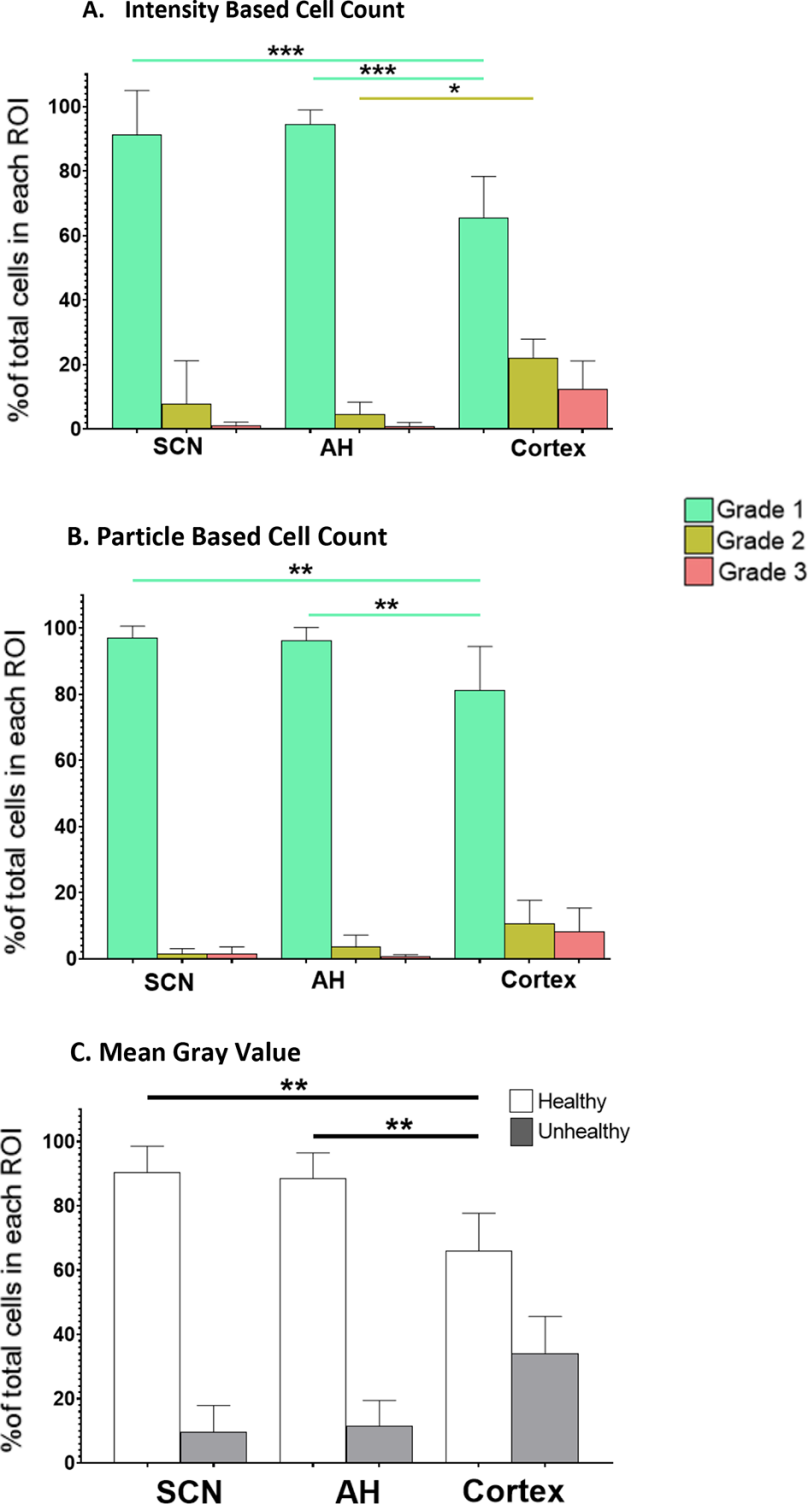
The percentage of healthy (Grade 1), damaged (Grade 2) and dead (Grade 3) cells in different brain slice regions. Differences under control conditions at ZT6 were analyzed using (A) the IBCC protocol or (B) the PACC protocol. (C) The percentage of healthy vs. unhealthy cells calculated using the MGV analysis protocol. * *p*<0.05; ** *p*<0.005; *** *p*<0.001. N=6 for each group (One-way ANOVA).

To confirm that PI imaging would be useful for assessing excitotoxic damage, tissue slices containing the different regions were left untreated or treated with 50 μM NMDA at ZT6. Figure 1 shows representative images of the different brain areas. The ROIs are marked by the white lines. Clear differences in overall PI staining are apparent, suggesting there are differences in tissue health across the brain regions under both control and NMDA-treated conditions. Based on these apparent differences, we proceeded to compare the extent of regional cell death and damage, beginning with control conditions, using all three of the analysis protocols. The data from these comparisons are summarized in Figure 2.

**IBCC**: Overall, IBCC analyses found no differences between the SCN and AH, but identified differences between these two areas and the cortex. More specifically, one-way ANOVA determined that there were no statistically significant differences in the percentage of Grade 1 (healthy), 2 (damaged) or 3 (dead) cells ( *p*=0.9) between the SCN and AH under control conditions. However, one-way ANOVA indicated a statistically significant difference between the SCN and cortex ( *p*<0.05). Post-hoc analysis determined that there was a statistically significant difference in the percentage of Grade 1 cells between SCN and cortex ( *p*=0.0001) but not for the percentage of Grade 2 ( *p*=0.0880) or Grade 3 ( *p*=0.2860) cells. One-way ANOVA also indicated a statistically significant difference between the AH and cortex ( *p*<0.05). Post-hoc analysis determined there was a statistically significant difference in Grade 1 cells ( *p*<0.0001) and Grade 2 cells ( *p*=0.0164) but not Grade 3 cells ( *p*=0.2860) between AH and cortex under control conditions.

**PACC**: Overall, PACC analyses also found no differences between the SCN and AH, but again found differences between these two areas and the cortex. More specifically, one-way ANOVA determined that there were no statistically significant differences in the percentage of Grade 1 (healthy), Grade 2 (damaged) or Grade 3 (dead) cells ( *p*=0.9) between the SCN and AH under control conditions. However, one-way ANOVA showed there was a statistically significant difference between the SCN and cortex ( *p*<0.05). Post-hoc analysis determined that there was a statistically significant difference in the percentage of Grade 1 cells between the SCN and cortex ( *p*=0.0012) but not Grade 2 ( *p*=0.1956) or Grade 3 ( *p*=0.6074) cells. One-way ANOVA also showed a statistically significant difference between the AH and cortex ( *p*<0.05). Post-hoc analysis determined that there was statistically significant difference in the percentage of Grade 1 cells between AH and cortex ( *p*=0.0027) but not Grade 2 ( *p*=0.5235) or Grade 3 ( *p*=0.4377) cells.

**MGV**: Similar to the IBCC and PACC analyses, MGV analysis found no differences between the SCN and AH, but identified differences between these two areas and the cortex. Speficially, one-way ANOVA determined there were no statistically significant differences in the percentage of Grade 1 (healthy) or Grade 2/3 (unhealthy) cells ( *p*=0.9) between the SCN and AH. However, one-way ANOVAs revealed statistically significant differences in Grade 1 (healthy) cells between the SCN and cortex ( *p*=0.0014) and between the AH and cortex ( *p*=0.0034). However, no significant differences in Grade 2/3 (unhealthy) cells were observed.

Thus, overall, we found that under control conditions the SCN and AH exhibit robust health, and that cortical slices exhibit more tissue damage compared to both the AH and SCN regions. The differences were largely restricted to significant decreases in the percentage of Grade 1 (healthy) cells in the cortex and some significant increases in the percentage of Grade 2 (damaged) cells, with no significant differences in the percentage of Grade 3 (dead) cells (based on IBCC and PACC analyses) across the three regions. Given the baseline differences in overall health of the cortex compared to the SCN and AH under control conditions, we determined that the effects of excitotoxic treatments should be assessed separately within each brain region rather than directly comparing across regions.

### Comparing the extent of excitotoxic damage using the different image analysis protocols

**Figure 3.**
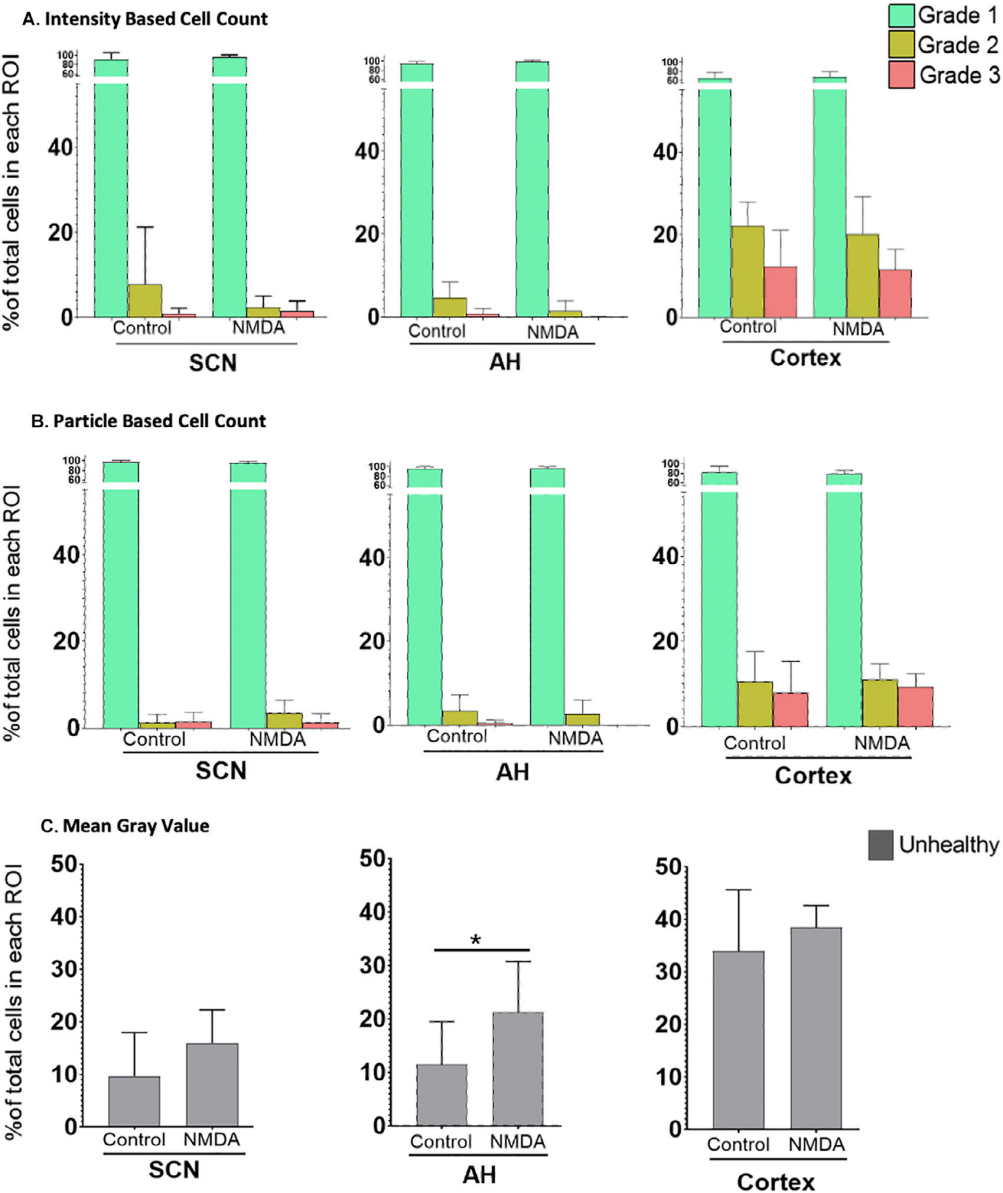
The percentage of healthy (Grade 1), damaged (Grade 2) and dead (Grade 3) cells in different regions of the brain. Differences under control *vs.* 50 μM NMDA treatment at ZT6 were analyzed using (A) the IBCC protocol or (B) the PACC protocol. (C) The percentage of unhealthy cells calculated using the MGV analysis protocol. * *p*<0.05; ** *p*<0.005; *** *p*<0.001. N=6 for each group (Student’s t-test). Note the change in Y axis scale compared to Figure 2 so that the percentages of damaged and dead cells are clearer.

Next, we sought to compare the three analysis protocols for their ability to assess differences in excitotoxic damage based on the intensity of PI staining in each region under control versus NMDA (50 μM) conditions. The results, based on the three image analysis protocols, are shown in Figure 3. Based on our experimental design, we focused on potential changes within the different cell Grades within each region across the different experimental conditions.

Using the IBCC analysis protocol, Student’s t-tests were used to test for changes in Grade 1 (healthy), Grade 2 (damaged) and Grade 3 (dead) cells separately within individual regions. There were no significant differences in the percentage of Grade 1 ( *p*=0.195), Grade 2 ( *p*=0.155) and Grade 3 ( *p*=0.314) cells in the SCN, in the AH [Grade 1 ( *p*=0.100), Grade 2 ( *p*=0.058) and Grade 3 ( *p*=0.059) cells] or in the cortex [Grade 1 ( *p*=0.354), Grade 2 ( *p*=0.337), and Grade 3 ( *p*=0.428) cells] when comparing control versus NMDA treated conditions within each brain region individually. Likewise, based on the PACC analysis procedure, Student’s t-tests determined there were no statistically significant differences in the percentage of Grade 1 ( *p*=0.229), Grade 2 ( *p*=0.067) and Grade 3 ( *p*=0.4613) cells in the SCN, in the AH [Grade 1 ( *p*=0.265), Grade 2 ( *p*=0.361) and Grade 3 ( *p*=0.063) cells] or in the cortex [Grade 1 ( *p*=0.345), Grade 2 ( *p*=0.431) and Grade 3 ( *p*=0.353) cells] when comparing control versus NMDA-treated conditions within each brain region individually. Thus, both IBCC and PACC analyses found no significant differences within brain regions in response to 50 μM NMDA treatment.

Using data from the MGV procedure, Student’s t-tests determined there were no statistically significant differences in the percentage of Grade 2/3 (unhealthy) cells in either the SCN ( *p*=0.072) or the cortex ( *p*=0.200) under control versus-NMDA treated conditions. However, Student’s t-tests determined there was a statistically significant difference in the unhealthy cells in the AH region between control and NMDA conditions ( *p*=0.041). These results, therefore, are somewhat different from those using the previous two procedures.

**Figure 4.**
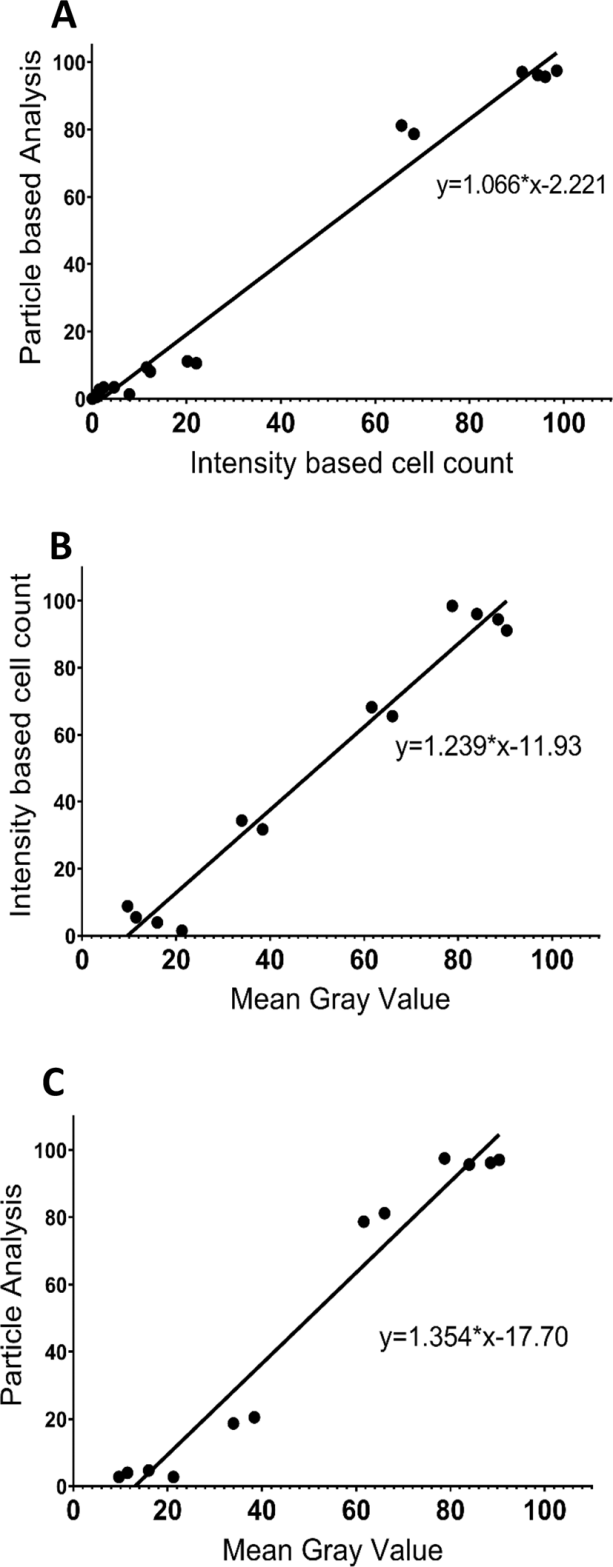
Correlations between the different image analysis procedures based on the results from 50 μM NMDA and control treatments at ZT6 for the three brain regions. (A) correlation between the PACC and IBCC protocols (R=0.9908, *p*<0.0001); (B) correlation between IBCC and MGV protocols (R=0.9825, *p*<0.0001); (C) correlation between MGV and PACC protocols (R=0.9791, *p*<0.0001). For B and C, Grade 2 and Grade 3 cell percentages determined using the IBCC and PACC protocols were combined to compare with unhealthy cells based on the MGV protocol, while Grade 1 cell percentages from the IBCC and PACC analyses were compared with the healthy cells based on the MGV protocol.

To further compare the three analysis procedures, we performed a Pearson Correlation Analysis for each pair of analysis protocols using both the control and 50 μM NMDA treatment data ( Figure 4). The results show that the three methodologies were highly correlated, with each pairwise comparison having an R value exceeding 0.98 and with *p*<0.0001. Thus, all three image analysis protocols appear to generate generally consistent data and are all useful for comparing the extent of PI staining across different tissues/conditions. However, although the MGV procedure is the most straightforward, it provides less detail than the other two methods, which are able to distinguish damaged versus dead cells. The PACC and IBCC methods also appear to be slightly more conservative than the MGV procedure in terms of assessing cell damage/death. Based on these results, for the remainder of the experiments we chose to analyze the images using the IBCC method only.

### The SCN exhibits greater dose-dependent resiliency to excitotoxic damage compared to the AH and cortex

**Figure 5.**
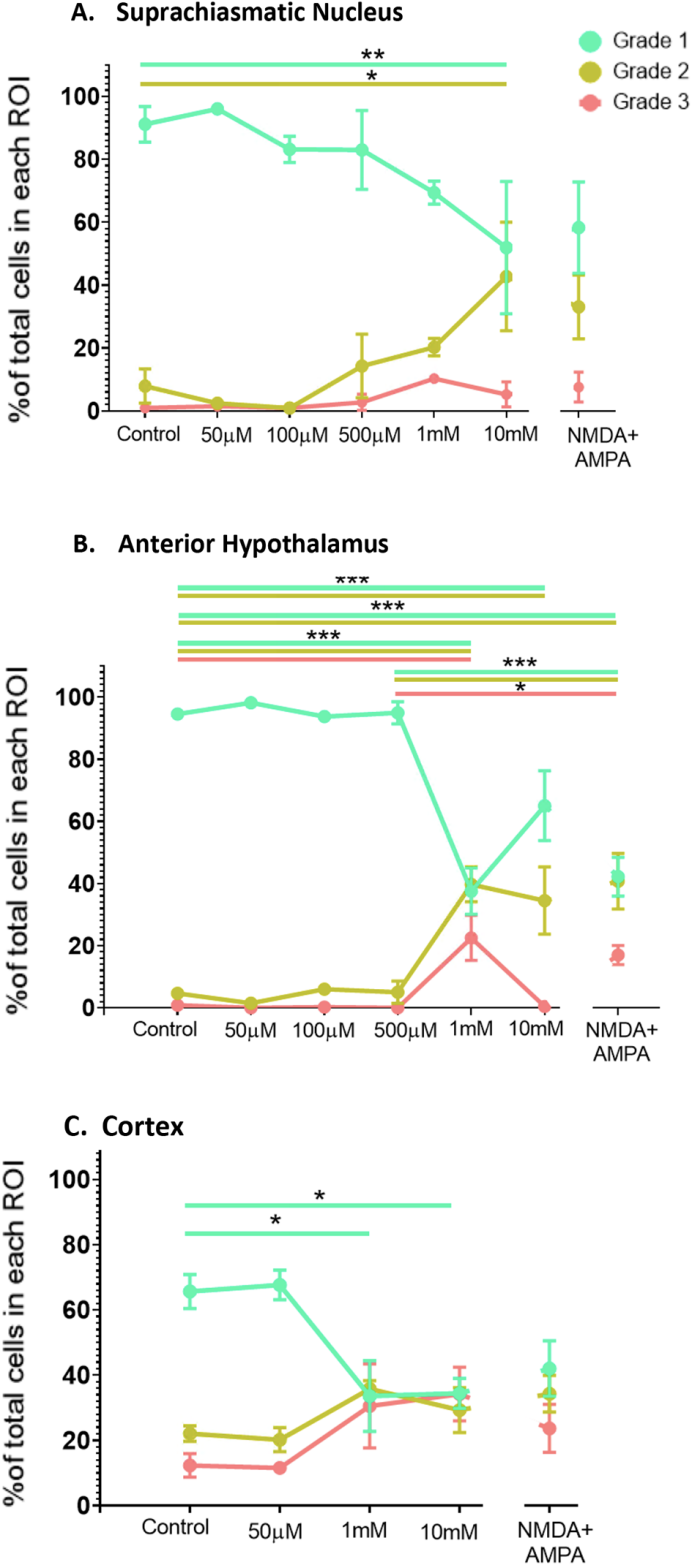
Dose response data for the SCN, the AH and the cortex exposed to increasing concentrations of NMDA (50 μM-10 mM) or NMDA (500 μM) + AMPA (50 μM) at ZT6. One-way ANOVA followed by Tukey’s post-hoc analysis revealed significant differences in the percentage of healthy (Grade 1), damaged (Grade 2) and/or dead (Grade 3) cells in the (A) SCN, (B) the AH and (C) cortex. * *p*<0.05; ** *p*<0.005; *** *p*<0.001. N=3-6 for each group.

Given the minimal effects of 50 μM NMDA treatment at ZT6 across all three brain regions, we proceeded to investigate whether more robust excitotoxic stimuli would increase cell damage when applied at this time point. For this, we treated the brain slices with increasing concentrations of NMDA (up to 10 mM) as well as with a combination of NMDA (500 μM) and AMPA (50 μM). The data are summarized in Figure 5.

In the SCN, one-way ANOVA determined that there were statistically significant differences across the treatment conditions ( *p*<0.05). Post-hoc analysis determined that there was a statistically significant difference in Grade 1 ( *p*=0.0432) and Grade 2 ( *p*=0.0221) cells between control and 10 mM NMDA conditions, but not when comparing control with lower concentrations of NMDA. Thus, at the highest concentration of NMDA we used there was a decrease in Grade 1 (healthy) cells and a concomitant increase in the percentage of Grade 2 (damaged) cells but not Grade 3 (dead) cells in the SCN.

In the AH, one-way ANOVA also showed statistically significant differences across the treatment conditions ( *p*<0.05). Post-hoc analysis determined there were statistically significant differences in Grade 1 (healthy) cells between control and 1 mM NMDA ( *p*<0.0001) and between control and 10 mM NMDA ( *p*=0.0042). For Grade 2 (damaged) cells there were statistically significant differences between control and 1 mM NMDA ( *p*=0.0001) and between control and 10 mM NMDA ( *p*=0.0024). For Grade 3 (dead) cells there was a statistically significant difference between control and 1 mM NMDA conditions ( *p*=0.0002) but no difference between control and 10 mM. Thus, both 1 mM and 10 mM NMDA treatments decreased the percentage of Grade 1 (healthy) cells and increased the percentage of Grade 2 (damaged)/Grade 3 (dead) and cells in the AH.

With respect to the cortex, one-way ANOVA indicated that there was a statistically significant difference across the experimental conditions ( *p*<0.05). Post-hoc analysis indicated there were statistically significant differences in the percentage of Grade 1 (healthy) cells between control and 1 mM NMDA ( *p*=0.0251) and between control and 10 mM NMDA ( *p*=0.0157). Thus, similar to the AH, both 1 mM and 10 mM NMDA treatments decreased the percentage of Grade 1 (healthy) cells and in the cortex.

One-way ANOVA determined that the combined treatment of NMDA (500 μM) + AMPA (50 μM) induced statistically significant changes in the AH compared to control ( *p*<0.05). Post-hoc analysis determined there were statistically significant differences in Grade 1 cells ( *p*<0.0001) and Grade 2 cells ( *p*=0.0003) in the AH region between control versus NMDA+AMPA conditions. Additionally, one-way ANOVA revealed statistically significant differences when comparing the response of the AH to 500 μM NMDA to that of NMDA (500 μM) + AMPA (50 μM). Post-hoc analysis determined that there were statistically significant differences between Grade 1 ( *p*=0.0001), Grade 2 ( *p*=0.0017) and Grade 3 ( *p*=0.0229). In contrast, one-way ANOVA determined that there were no significant differences in the SCN region under control versus NMDA+AMPA conditions ( *p*>0.05). Similarly, NMDA+AMPA did not induce statistically significant changes in the cortex compared to control ( *p*>0.05). There also were no statistically significant differences in the SCN when comparing the response to 500 μM NMDA versus NMDA (500 μM) + AMPA (50 μM). Such a comparison, however, was not possible for the cortex because we did not treat cortical slices with 500 μM NMDA alone.

### The SCN, AH and cortex are more susceptible to excitotoxic damage during the night than during the day

**Figure 6.**
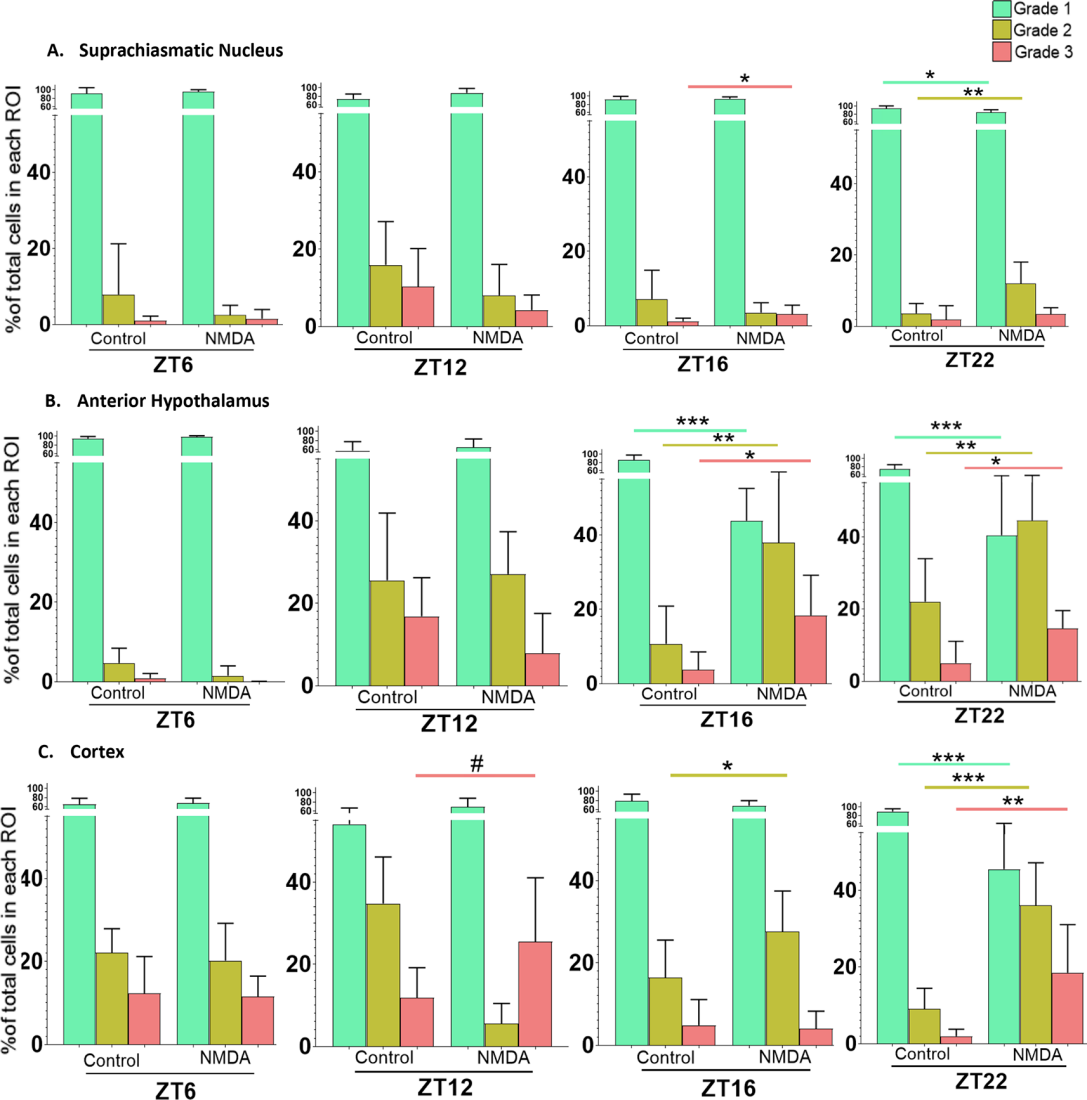
Time of day differences in excitotoxic responses in the SCN, AH and cortex treated with 50 μM NMDA. Non-paired student t-tests between control and treated samples at different treatment times (ZT6, ZT12, ZT16 and ZT22) revealed significant differences in the percentage of healthy (Grade 1), damaged (Grade 2) and/or dead (Grade 3) cells. * *p*<0.05; ** *p*<0.005; *** *p*<0.001, # p approaching significance. N=6 for each group.

To determine whether excitotoxic susceptibility exhibits diurnal variations, tissue slices containing the different brain regions were left untreated or treated for one h with 50 μM NMDA at ZT6, 12, 16 or 22. Because preparing brain slices during the lights-off period can reset the circadian clock, ^11^ we prepared all the slices in the lights-on period. Due to this constraint, treating the slices at four different ZTs required that they be maintained *in vitro* for different amounts of time. To account for this, slices treated at ZT6 and ZT16 were prepared four to six hours prior to treatment, while slices treated at ZT12 and ZT22 were prepared 10-12 hours prior to treatment. Again, because we were investigating changes in the percentage of cells within each Grade in individual regions in response to treatments at different times of day, we used Student’s t-tests for the statistical analyses. The data are summarized in Figure 6.

In the SCN treated at ZT6 and ZT12, Student’s t-tests determined there were no statistically significant differences between control and NMDA-treated conditions [Grade 1 (healthy) ( *p*=0.026; not considered significant because the trend was in the opposite direction from the one-tailed test), Grade 2 (damaged) ( *p*=0.096), and Grade 3 (dead) ( *p*=0.94) cells]. However, with the SCN treated at ZT16, Student’s t-tests revealed a statistically significant difference between control and NMDA treated slices in the percentage of Grade 3 cells ( *p*=0.047) but not for Grade 1 ( *p*=0.326) or Grade 2 ( *p*=0.150) cells. For the SCN treated at ZT22, Student’s t-tests revealed statistically significant differences in the percentage of Grade 1 ( *p*=0.009) and Grade 2 ( *p*=0.005) cells between control and NMDA treated conditions but no statistically significant difference between Grade 3 cells under the two conditions ( *p*=0.205). Taken together, these data suggest that SCN is more susceptible to excitotoxic damage during the middle and late night than during the day, with an increase in dead cells only occurring after treatment at ZT 16.

For the AH treated at ZT6 and ZT12, Student’s t-tests determined that there were no statistically significant differences between control and NMDA conditions [Grade 1 (healthy) ( *p*=0.263), Grade 2 (damaged) ( *p*=0.427) and Grade 3 (dead) ( *p*=0.067) cells]. However, for the AH treated at ZT16, Student’s t-tests indicated statistically significant differences in the percentage of Grade 1 ( *p*=0.00002), Grade 2 ( *p*=0.005) and Grade 3 ( *p*=0.006) cells between control and NMDA treated conditions. A similar pattern was observed for the AH treated at ZT22, where Student’s t-tests indicated statistically significant differences in Grade 1 ( *p*=0.0009), Grade 2 ( *p*=0.005) and Grade 3 ( *p*=0.006) cells between control and NMDA-treated conditions. Thus, the AH is also more susceptible to excitotoxic damage during the night versus the day. Moreover, the degree of excitotoxic damage appears to be more substantial in the AH versus the SCN, based on significant decreases in Grade 1 cells and increases in Grade 2 and Grade 3 cells in the AH after treatment at both ZT 16 and 22.

For cortical brain slices treated at ZT6, Student’s t-test indicated that there were no statistically significant differences in the percentage of Grade 1 (healthy) ( *p*=0.389), Grade 2 (damaged) ( *p*=0.337) or Grade 3 (dead) ( *p*=0.428) cells between control versus NMDA conditions. For cortical slices treated at ZT12, Student’s t-test indicated that there was a difference in the percentage of Grade 3 cells that did not quite reach significance ( *p*=0.0541) between control vs NMDA conditions. For cortical slices treated at ZT16, Student’s t-test indicated a statistically significant difference in the percentage of Grade 2 cells ( *p*=0.034) under NMDA-treated conditions compared to control conditions but not Grade 1 ( *p*=0.103) or Grade 3 ( *p*=0.395) cells. Lastly for cortical slices treated at ZT22, Student’s t-test indicated statistically significant differences in the percentage of Grade 1 ( *p*=0.00002), Grade 2 ( *p*=0.00007) and Grade 3 ( *p*=0.0039) cells between control and NMDA treated conditions. Thus, the cortex is also more susceptible to excitotoxic damage during the night versus the day. Further, the degree of excitotoxic damage again appears to be more substantial compared to the SCN based on the percentages of healthy, damaged, and dead cells as well as possibly the range of times when it exhibits greater excitotoxic susceptibility.

## Discussion

Although previous studies have reported that the SCN is resilient to excitotoxic stimuli, neither its degree of resilience compared to other brain areas nor whether there are diurnal variations in its resiliency have been investigated previously. Using acute brain slices, which preserve the *in vivo* cytoarchitecture, we assessed differences in excitotoxic susceptibility across multiple brain regions and time points and tested varying strengths of excitotoxic stimulation using PI as a cell death/damage marker. The extent of PI incorporation into neurons has been correlated with functional changes, including the ability to maintain normal resting membrane potentials and to generate action potentials. ^10^ Together, our data demonstrate that the SCN is more resistant to NMDA-induced excitotoxicity compared to the AH and the cortex. The data also show that there are time-of-day differences in excitotoxic resiliency, with the extent and pattern of excitotoxic susceptibility differing across each region.

We began with comparing the general health of the cells within brain slices containing each brain region under control conditions. This was done to distinguish differences due intrinsic and/or slice preparation factors versus differences in excitotoxic resiliency. Using three image analysis protocols, we determined that under our control conditions cell damage in cortical brain slices was greater than in either the SCN or the AH. This could be due to differences in neuronal phenotype, the extent of damage to fibers of passage, or other factors. Regardless, the differences under baseline conditions required us to analyze the excitotoxic responses in each brain region separately.

### Regional differences in excitotoxic susceptibility

We initially assessed excitotoxic susceptibility by determining the effect of 50 μM NMDA applied to the brain slices at ZT6, maintaining the tissues for a total of 6 h after the onset of NMDA treatment, and 5 h after the end of the NMDA treatment. This post-treatment interval was implemented to allow time for initial cellular changes to unfold, whether they involved recovery or damage/death processes. We again analyzed the data using three image analysis protocols in order to compare their utility. Using the MGV analysis, which only distinguishes between healthy (Grade 1) versus unhealthy (Grade 2+3) cells, we found a significant increase in unhealthy cells in the AH but not in the SCN or the cortex in response to 50 μM NMDA. These data are consistent with those from previous studies. Peterson *et al.* ^6^ showed that kainic acid induces more cell damage in hypothalamic areas surrounding the SCN versus the SCN itself, although it is possible that the regional differences were influenced by the extent of kainic acid diffusion through the tissue. Separately, Bottum *et al.* ^7^ used cultures of immortalized cells to show that glutamate induces more cell death in GT1-7 (hypothalamic) cells than SCN2.2 cells. However, results using immortalized cells in culture could differ from either *in vivo* or *ex vivo* preparations due to the loss of the intact tissue environment. ^12^ Importantly, similarly to the MGV analysis protocol, both of these previous studies utilized analyses that would not necessarily distinguish between dead and damaged cells.

On the other hand, the IBCC and PACC analyses, which distinguish between healthy, damaged, and dead cells, did not show significant increases in excitotoxic damage in any of the three regions in response to 50 μM NMDA. Despite the different results across the three analyses, Pearson Correlation analysis confirmed that the data generated by all three protocols were highly correlated. Nevertheless, comparing the results obtained using the three protocols suggests that the more nuanced analyses (IBCC and PACC) provide a different, albeit perhaps more conservative, assessment of the degree of excitotoxic damage inflicted on cells in our brain slices compared to the MGV analysis. Based on these differences, we decided to use the IBCC analysis protocol for the remainder of the study. Nevertheless, in future studies of this nature a further exploration of analytical methods could potentially identify advantages/disadvantages of different approaches.

Our dose-response experiments, where we treated the slices with increasing concentrations of NMDA, confirm and build upon the enhanced excitotoxic resiliency of the SCN shown in previous studies. Specifically, we showed that the AH and the cortex exhibited a significant decrease in the percentage of healthy cells in response to both 1 mM and 10 mM NMDA compared to control conditions, with the AH also showing significant increases in damaged and dead cells in response to 1 mM NMDA. The SCN, on the other hand, only exhibits decreases in healthy cells and increases in damaged cells in response to the highest concentration of NMDA used (10 mM). The differences between the SCN and AH are notable given that the data are generated from the same brain slices, and from adjacent areas within the brain slices ( *e.g.*, see Figure 1). Thus, differences in slice preparation, tissue handling, or other factors that could vary between individual slices cannot account for these differences. Although we did not test cortical slices with 100 or 500 mM NMDA, the substantial cell death/damage seen in response to 1 mM NMDA is clearly different from that seen in the SCN. If anything, such data would show an even greater susceptibility of the cortex to excitotoxic stimulation.

The lack of significant increases in dead or damaged cells in the cortex while there were significant decreases in healthy cells could be due to the percentage of both Grade 2 and Grade 3 cells appearing to increase to similar extents (see Figure 5), such that neither change by itself reaches significance. This pattern is different from that seen in both the SCN and AH, where the percentage of dead cells consistently appeared lower than the percentage of damaged cells. Moreover, the percentage of Grade 1 cells in the cortex decreased to the same level as that of Grade 2 and Grade 3 cells in response to both 1 mM and 10 mM NMDA, consistent with substantial cell death/damage in the cortex under these stronger excitotoxic conditions. Again, this differs substantially from the pattern seen in the SCN. Taken together, the results are consistent with the SCN having at least a 10-fold greater resiliency to NMDA-induced excitotoxicity compared to the AH and cortex.

In addition to treating brain slices with increasing concentrations of NMDA, we also treated them with a combination of NMDA and AMPA. This was done because AMPA receptor activation can enhance NMDA receptor signaling by removing the Mg ^2+^ block. ^13^ We determined that combining 50 μM AMPA with 500 μM NMDA did not exacerbate cell damage in the SCN or cortex beyond that seen in response to control or (for SCN) 500 μM NMDA alone, but it significantly increased the percentage of damaged cells in the AH compared to both control and 500 μM NMDA alone. It is possible that lack of an additive effect of AMPA in the SCN was due to the neurons in this region already having a relatively high resting membrane potential. ^14^ The fact that cell damage did not increase significantly in the cortex in response to NMDA+AMPA compared to control conditions again could be, as seen in response to 1 mM and 10 mM NMDA, because the percentages of both Grade 2 and Grade 3 cells appeared to increase to similar extents to levels comparable to that of Grade 1 cells, so that the changes within each cell Grade did not reach significance.

Although in this study we have compared the excitotoxic susceptibility of the SCN to the surrounding hypothalamus and the cortex, there are other data showing regional differences in excitotoxic effects. For example, the CA2 region of the hippocampus has been reported to be more resistant to cell damage than other areas of the hippocampus in both human and animal models of hypoxia and traumatic brain injury. ^15^ Additionally, retinal ganglion cells have also been reported to be resistant to NMDA toxicity both in *in vitro* and *in situ* models. ^16^ It would be interesting in future studies to compare these two regions, together with the SCN, to potentially explore their distinct responses to excitotoxic challenges. 

### Possible mechanisms of excitotoxic resiliency

Although excitotoxicity involves multiple cellular process, including mitochondrial dysfunction and increased generation of reactive oxygen species, ^17^ it generally is initiated by excessive glutamate stimulation followed by a surge in intracellular Ca ^2+^. Since, across the different types of glutamate receptors, NMDA receptors typically have the highest Ca ^2+^ permeability, ^18^^,^^19^ they are a primary factor driving excitotoxic damage. In this regard, heterogeneity in NMDA receptor expression and subunit composition could contribute to regional differences in the observed excitotoxic susceptibility. In particular, GluN2B subunit expression has been associated with increased excitotoxicity-induced neuronal apoptosis, while GluN2A subunit expression is associated with enhanced neuronal survival in both *in vitro* and *ex vivo* models of ischemic stroke. ^20^ Bottum *et al.* (2010) found in cell culture studies that *GluN2A* mRNA was expressed at higher levels in SCN2.2 cells compared to GT1-7 cells. ^7^ These data are consistent with an increased resiliency of the SCN; however, it will be important to determine if the differences in mRNA translate to similar differences in the protein levels, and to assess whether these differences are also seen under *in vivo* and *ex vivo* conditions. Bottum *et al.* also found that SCN2.2 cells express *GluN2B* mRNA which was undetectable in GT1-7 cells. ^7^ This is unexpected, given that GluN2B subunit expression is associated with increased excitotoxicity-induced apoptosis, ^20^ although GluNR2B-containing NMDA receptors may play a neuroprotective role in developing hippocampal neurons. ^21^ These differences regarding a role for GluNR2B in excitotoxicity might be due to different subunit locations (extra-synaptic region versus the synaptic) and their interactions with intracellular proteins. ^22^^–^^24^ With respect to cortical excitotoxic susceptibility, both *GluN2A* and *GluN2B* mRNA are expressed throughout the forebrain in both mice and rats in levels that vary across age. ^19^^,^^20^ However, the relative percentages of these two subunits compared to the hypothalamus have not been studied. Thus, any contribution that NR2B and NR2B subunit expression have on the differential resiliency to excitotoxicity seen in this study remains to be elucidated.

It is interesting to speculate as to whether the enhanced excitotoxic resiliency of the SCN is important because the SCN receives a large amount of glutamate stimulation from retinal ganglion cells in response to light, and therefore needs compensatory mechanisms. ^25^ As noted above, a key factor in excitotoxicity is a surge in intracellular Ca ^2+^. If not quickly buffered, the increase in cytoplasmic Ca ^2+^ can damage intracellular organelles and activate Ca ^2+^-dependent cell death pathways. ^26^ Cells that can effectively buffer Ca ^2+^ surges might exhibit greater resiliency to excitotoxic damage. In this regard, the SCN contains a dense population of cells expressing the Ca ^2+^ binding protein calbindin. ^27^ It also expresses another Ca ^2+^ binding protein, calreticulin, although to a lesser extent. ^28^ On the other hand, the SCN expresses lower levels of the Ca ^2+^ binding protein parvalbumin, which is more abundant in the cortex. ^29^ The differential expression of these proteins might enhance the Ca ^2+^ buffering capacity of the SCN cells, although this would need to be explored in future studies.

### Time-of-day differences in excitotoxic susceptibility

In addition to showing regional differences in NMDA-induced excitotoxic susceptibility, our study also determined that excitotoxic susceptibility within each region followed a diurnal rhythm. In all three regions, 50 μM NMDA decreased the percentage of healthy cells and/or increased the percentage of damaged/dead cells during the night more than during the day. In line with the resiliency shown in the dose-response experiments, the excitotoxic effects in the SCN again were less than those seen in both the AH and cortex, both in terms of the range of times that excitotoxic effects are seen as well and number of timepoints when significant increases in cell death occur. Several studies have investigated in vivo diurnal rhythms in response to ischemia or traumatic brain injury (TBI). Two studies showed larger infarct volume after ischemia induced prior to or the beginning of the light period compared to late in the subjective day or middle of the dark period. ^30^^,^^31^ Two other studies found greater neurological deficits and cell damage after TBI induced during the early or middle of subjective day vs. mid- to late subjective night. ^32^^,^^33^ Conversely, more cell death was observed in the hippocampus in response to nighttime compared to daytime ischemia. ^34^ The differences across these studies could be due to a variety of methodological factors, including differences in brain regions, methods of trauma, and how damage was assessed. It is thus noteworthy that is the first time a rhythm in excitotoxic susceptibility has been identified in the cortex, SCN and AH under these well-controlled conditions.

It is not surprising that the SCN exhibits a daily rhythm in excitotoxic resiliency, given that it is the site of the primary mammalian circadian oscillator, and as such it expresses circadian rhythms in numerous cellular processes. ^35^ The rhythm in excitotoxic resiliency could be due to a variety of previously characterized processes, including rhythms in NMDA receptor composition and activity. Studies have shown that *GluNR2B* mRNA expression in the SCN is higher at ZT10 and ZT16 compared to other time points, and phosphorylation of GluN2B subunits, which increases their activity, is highest at ZT20. ^36^ Additionally, the NMDA component of post-synaptic potentials recorded in the SCN in response to optic nerve stimulation are larger in the night compared to the day, consistent with NMDA activity being required for phase shifting of the clock in the night. ^37^^,^^38^ Also, NMDA-induced Ca ^2+^ transients in the SCN are more robust in response to bath application of NMDA at night compared to the day. ^39^ Thus, greater NMDA receptor activity (and increases in intracellular Ca ^2+^) in the SCN at night could contribute to greater nighttime excitotoxic responses in the SCN. Along with this, decreased nighttime calbindin expression, and therefore decreased Ca ^2+^ buffering, could also increase excitotoxic susceptibility at night. ^40^

It is also possible that time-of-day differences in SCN excitotoxic susceptibility involve glial cells. Astrocytes in the SCN have daily rhythms of glial fibrillary acid protein (GFAP) expression, synapse-associated morphology, ATP release and glutamate release. ^41^^–^^44^ Decreased astrocytic arborization and synaptic proximity concurrent with increased astrocytic glutamate release at night could potentially raise the extracellular glutamate concentration, which could increase excitotoxic susceptibility. However, such a possibility would need to be investigated further.

Shifting to the AH and cortex, given that many extra-SCN brain regions have also been shown to exhibit circadian rhythms in cellular activity ^45^ it is not surprising that the AH and cortex also exhibited rhythms in excitotoxic resiliency. However, generally brain regions outside the SCN exhibit rhythms have a reversed phase compared to the SCN. ^46^^,^^47^ Thus, it is surprising that all three areas investigated in this study exhibit a similar phase (more at night versus the middle of the day) in rhythmic excitotoxic susceptibility. Importantly, the staggered times of brain slice preparation we used in these experiments eliminates time *in vitro* as an explanation for this rhythm in excitotoxic susceptibility. The mechanisms underlying the rhythms in the AH and cortex could be the same or distinct from those in the SCN. However, the near synchrony in excitotoxic susceptibility across all three regions suggests that exploring aspects of cellular physiology that exhibit similar phases across these three brain regions could prove fruitful in the search for mechanisms contributing to excitotoxic resiliency.

### Limitations of the study

There are several limitations of this study. First, we did not investigate the critical issue of whether the time of day differences we observed are regulated by the circadian clock or whether they are the result of the light-dark environment. Subsequent investigations can address this by repeating the experiments in tissue collected from animals housed in constant lighting conditions. Second, we only analyzed the effects of a single duration of excitotoxic stimulation (1 h) followed by a total of 5 h post-stimulus prior to tissue fixation. As noted, we chose these parameters so we could assess tissue responses proximal to the excitotoxic stimulus. Although the use of acute brain slices limits experimental durations to some extent, it would be interesting to assess longer excitotoxic exposures as well as longer post-exposure periods. For example, we have performed similar experiments exposing SCN and cortex slices to amyloid oligomers for 4 h during the daytime, followed by 2 of post-exposure perfusion prior to fixation and found significant increases in cell death and damage. ^48^ Third, we did not parse our analyses to investigate subregions within the cortex or SCN. As illustrated in Figure 1, our ROIs encompassed the entire coronal aspect of the SCN and included all cortical sublayers contained in our histological sections. Thus, exploring cell-type differences in excitotoxic susceptibility within each region could provide insight into potential cell-type differences. Fourth, we only used PI incorporation to assess the extent of cell damage. Using additional methods such as lactate dehydrogenase release or apoptotic marker expression could provide additional information into the cellular processes occurring in response to the excitotoxic stimulation. Lastly, we only used tissues from young adult male mice for these experiments. Comparing the effects across sex and age could again provide important clinically relevant information.

In summary, we have confirmed and extended previous reports indicating that the SCN is more resilient to excitotoxic damage compared to other brain regions. The excitotoxic susceptibility in all three brain regions investigated is dose-dependent. However, compared to the AH and cortex, a ten-fold higher concentration of NMDA applied at ZT 6 was needed to increase cellular damage in the SCN, and even at the highest dose we tested there were no statistically significant increases in cell death in the SCN. We have also demonstrated, for the first time, that in the SCN (and other brain regions) excitotoxic susceptibility is dependent on the time-of-day when the stimulus is applied, with greater susceptibility occurring during the night period. However, consistent with the greater dose-dependent resiliency seen in the SCN, an increase in cell death was only observed in the SCN after treatment at ZT 16, and an increase in cell damage was only seen in the SCN after treatment at ZT 22, while both the AH and cortex exhibited decreases in healthy cells and increases in cell death and damage across a broader range of treatment times. As discussed, the increased resiliency of the SCN could be due to a variety of factors. Future studies identifying these neuroprotective mechanisms could help in developing therapies aimed at treating conditions linked to excitotoxic damage, including neurodegenerative diseases, stroke, and traumatic brain injury.

## Data Availability

Tennessee Research and Creative Exchange: ex vivo Comparative Investigation of Suprachiasmatic Nucleus Excitotoxic Resiliency - ex vivo Comparative Investigation of Suprachiasmatic Nucleus Excitotoxic Resiliency, https://doi.org/10.7290/xSDiunNFHq. ^49^ This project contains the following underlying data:
-
Prosser_DATASET_2022-08-15.txt -
Figure 2A_Raw Data_Manuscript.xlsx -
Figure 2B_Raw Data_Manuscript.xlsx -
Figure 2C_Raw Data_Manuscript.xlsx -
Figure 3A_Raw Data_Manuscript.xlsx -
Figure 3B_Raw Data_Manuscript.xlsx -
Figure 3C_Raw Data_Manuscript.xlsx -
Figure 5A_Raw Data_Manuscript.xlsx -
Figure 5B_Raw Data_Manuscript.xlsx -
Figure 5C_Raw Data_Manuscript.xlsx -
Figure 6A_Raw Data_Manuscript.xlsx -
Figure 6C_Raw Data_Manuscript.xlsx -
Figure 6C.xlsx Tennessee Research and Creative Exchange: ex vivo Comparative Investigation of Suprachiasmatic Nucleus Excitotoxic Resiliency - ex vivo Comparative Investigation of Suprachiasmatic Nucleus Excitotoxic Resiliency, https://doi.org/10.7290/xSDiunNFHq. ^49^ This project contains the following extended data:
- Tif imaging files for controls and treatments - Annotation for figures.docx Data are available under the terms of the Creative Commons Zero “No rights reserved” data waiver (CC0 1.0 Public domain dedication).
