# Supplementary material for: Ex vivo comparative investigation of suprachiasmatic nucleus excitotoxic resiliency
Source: F1000Res. 2023 Apr 26;11:1242. Originally published 2022 Nov 1. [Version 3] doi: 10.12688/f1000research.125332.3 (PMC11809682; doi:10.12688/f1000research.125332.3)
Supplement: Peer review history

# Reviewer report: Reviewer response for version 3

**DOI:** 10.5256/f1000research.146652.r345578

**Published:** 2024-12-16

**Authors:** Tak Pan Wong

**Affiliations:** 1 Douglas Hospital Research Centre, Douglas Mental Health University Institute, McGill University, Montreal, Canada

Acharyya et al in this study used brain slice to examine the vulnerability of neurons in suprachiasmatic nucleus (SCN), anterior hypothalamus (AH), and cortex (CC) from mice to excitotoxicity. Previously findings using in vivo or cultured neurons settings have suggested a higher resilience of SCN neurons towards excitotoxicity. However, limitations came from these settings raised questions of whether the higher resilience of SCN neurons was due to differential regional glutamate exposure in vivo or the lack of natural neural environment in culture cells. Using brain slices from male adult C57 mice, they estimated the density of unhealthy and damaged neurons in SCN, AH, and CC using propidium iodide (PI) staining after treating cells with different concentration of NMDA and AMPA. In addition, they examined excitotoxicity in slices at different times of the day (zeitgeber time (ZT) 6, 12, 16 or 22 hours after light on) after treating them with NMDA or with AMPA to induce excitotoxicity. Using 3 methods to quantify PI staining, they concluded that SCN neurons showed a higher resilience towards NMDA than AH and CC at a concentration dependent manner. They also found that while brain regions are generally more susceptible to excitotoxicity at later ZT, SCN neurons remain more resilient to NMDA treatments than AH and CC neurons. They concluded that SCN exhibits excitotoxic resiliency than AH and CC.

 Since excitotoxicity has been implicated in stroke and neurodegenerative diseases, understanding mechanisms underlying the vulnerability to excitotoxicity could reveal mechanisms for ameliorating excitotoxic damages. Regional differences in the vulnerability to excitotoxicity in the brain is also well known, like the CA2 and SCN. The current study was carefully designed. For instance, the comparison of SCN vs. AH in the same slice is a nice design to address the potential effects of slice differences. Data analyses were clearly described. Three methods of quantifying PI staining were used. The fact that findings from these methods correlated with each other is a strength of the study. The attempt to examine the impact of daytime vs. nighttime on excitotoxicity is interesting.

 Like previous findings, data from this study support the notion that SCN is more resistance to excitotoxicity. The lack of further investigation of mechanisms underlying the heightened mechanisms of SCN neurons remains a weakness of the current study. While the authors showed that SCN exhibited higher excitotoxicity resistance even at nighttime that other brain regions, it remains unclear what makes SCN more resistance to NMDA and or AMPA treatment. Having said that, potential mechanisms have been extensively discussed. Further studies could examine which of these mechanisms are responsible for the heightened resilience to excitotoxicity in SCN.

 Comparing the effects of day or and nighttime on excitotoxicity is a good start to reveal the impact of circadian rhythms on excitotoxicity. Since animals were sacrificed at different times of the day to maintain a similar incubation time for slices for daytime or nighttime examination, could the condition of animals at different time of the day relate to their susceptibility to excitotoxicity? For instance, corticosterone levels, which may affect to neuronal survival, fluctuate from daytime to nighttime daily. Other hormonal and physiological signals that exhibit circadian rhythms may also be responsible.

Is the work clearly and accurately presented and does it cite the current literature?

Yes

If applicable, is the statistical analysis and its interpretation appropriate?

Yes

Are all the source data underlying the results available to ensure full reproducibility?

Yes

Is the study design appropriate and is the work technically sound?

Yes

Are the conclusions drawn adequately supported by the results?

Yes

Are sufficient details of methods and analysis provided to allow replication by others?

Yes

Reviewer Expertise:

The impact of stress on synaptic and cognitive functions.

I confirm that I have read this submission and believe that I have an appropriate level of expertise to confirm that it is of an acceptable scientific standard.

# Reviewer report: Reviewer response for version 2

**DOI:** 10.5256/f1000research.142853.r159514

**Published:** 2023-02-24

**Authors:** Malgorzata Beresewicz-Haller

**Affiliations:** 1 Mossakowski Medical Research Institute, Polish Academy of Sciences, Warsaw, Poland

While the authors have addressed all the issues I raised, I cannot unfortunately claim to be fully satisfied with all the responses.

1. An analysis of Figures 3 and 5 may encourage a conclusion to the effect that, under excitotoxic stress, the suprachiasmatic nucleus is more resilient than the anterior hypothalamus. I would only speculate whether that is actually the case by 10-fold, but there is no evidence of differences in sensitivity between the suprachiasmatic nucleus and the cortex. The data described indicate that, under the influence of different concentrations of NMDA, or where NMDA and AMPA act simultaneously, the response is very similar at the two locations. Thus, 1mM NMDA does not cause statistically significant changes in Grades 2 and 3 (damaged or dead cells), in either the cortex (Figure 5C) or the suprachiasmatic nucleus (Figure 5A). In addition, 10mM NMDA causes a significant increase in Grade 2 (cell damage) in the suprachiasmatic nucleus, even as there is no effect in the cortex, and no change in Grade 3 (dead cells) in any region. Indeed, in both brain regions, there is a significant reduction of so-called healthy cells (Grade 1) under NMDA treatment, but not enough to convince me as to the adequacy of an argument referring to the regions manifesting differential sensitivity. In essence, therefore, any invoking of such differential sensitivity between the suprachiasmatic nucleus and cortex would have to be viewed as over-interpretation.
2. Going further, it is necessary to address the authors’ explanation as to why cell damage did not increase significantly in the cortex in response to NMDA alone or NMDA+AMPA, as compared with the controls. Specifically, they assert that this may reflect the way in which " *the percentages of both Grade-2 and Grade-3 cells appeared to increase at similar rates to levels comparable with Grade-1 cells, such that changes within each Grade did not reach significance*". Does this mean that an inappropriate image analysis protocol was chosen to analyse the data? Would use of the Mean Grey Value method not in fact have been a better solution here?
3. Again, I raise an issue to the effect that, under the conditions described in the manuscript, 50uM NMDA is not an appropriate concentration through which to determine the efficacy of the three different image analysis protocols. For, at that concentration, the excitotoxic effect is hardly noticeable, with only the Mean Grey Value method revealing an increase in neuronal death in the anterior hypothalamus. Hence, in the absence of differences between control and NMDA-treated conditions, Pearson's linear correlation analysis showed a high correlation coefficient for all comparisons; and did not differentiate between the tested image analysis protocols where there effectiveness was compared. In my view, the consequence of this has been that the choice of image analysis protocol was made on a basis hard for the reader to judge. In such a situation, I recommend very much that the analysis also be done for higher concentrations (e.g. 100 or 500uM) of NMDA, with correlation analysis again carried out. As these concentrations were used in another experiment, it is a matter of performing an analysis of images already taken, not additional experiments. However, should the new image analysis and re-correlation fail to point to the effectiveness of either protocol, please describe the rationale for your choice.
4. With changes now made to Figure 3C, it has become obvious what it illustrates. However, it is not clear to me why the pattern of data presentation in Figure 3C is different from that in Figures 3A and 3B – a circumstance that is confusing on the one hand, and also a source of difficulty when it comes to any comparison of the effectiveness of the three different image analysis protocols. Figures 3A and 3B show the distribution of PI intensity where the division is into three categories (Grades 1, 2 and 3) in the suprachiasmatic nucleus and anterior hypothalamus, under control and NMDA-treated conditions. Figure 3C only shows the data for Grades 2 and 3, in both control and NMDA-treated conditions within the cortex.

Is the work clearly and accurately presented and does it cite the current literature?

Yes

If applicable, is the statistical analysis and its interpretation appropriate?

Yes

Are all the source data underlying the results available to ensure full reproducibility?

Yes

Is the study design appropriate and is the work technically sound?

Yes

Are the conclusions drawn adequately supported by the results?

Partly

Are sufficient details of methods and analysis provided to allow replication by others?

Yes

Reviewer Expertise:

NA

I confirm that I have read this submission and believe that I have an appropriate level of expertise to confirm that it is of an acceptable scientific standard, however I have significant reservations, as outlined above.

# Response

**Published:** 2023-04-05

**Authors:** Rebecca Prosser

**Affiliations:** University of Tennessee Knoxville, USA

We appreciate the reviewer’s careful evaluation of our results, and we take to heart her thoughts regarding whether using the MGV procedure might be better than the IBCC procedure. Moreover, we realized that regardless of whether one is better than the other, they can each provide a different analytical perspective. Because it was the dose-response data that was of most concern, we re-analyzed the histology images for those data using the MGV procedure. However, rather than repeating what would essentially be the same test (whether there are significant changes within each cell category across experimental condition), we shifted to directly testing whether there were significant differences in the percentage of healthy vs. unhealthy cells across experimental conditions. In this case, significant differences indicate overall healthier conditions, while non-significant differences in the percentage of healthy and unhealthy cells (or a significantly higher percentage of unhealthy cells compared to healthy cells) indicate less healthy conditions. The results are largely consistent with the IBCC data. In fact, consistent with our previous conclusion that the MGV procedure generates less conservative data, the MGV data show additional differences when comparing the SCN to both the AH and cortex. Using MGV, the AH is only healthy under control, 50 uM and 100 uM NMDA conditions, while the cortex is only healthy under control and 50 uM NMDA conditions. The SCN, on the other hand, remains healthy up through 1 mM NMDA.

 Analysis of the MGV data indicated that AMPA+NMDA decreases tissue health in all three brain regions, which differs from the IBCC data that indicated effects of AMPA+NMDA only in the AH. This is certainly easier to explain and more consistent with our initial expectations, but we believe both sets of analyses need to be considered when interpreting the results overall.

 We have added the MGV data to Figure 5 to help with all of these comparisons, and we have included a discussion of the new data in the discussion section.

 Another point raised by the reviewer focuses on why we chose IBCC over the other two procedures. Although we alluded to previous data supporting its usefulness in evaluating PI staining differences, we have added text to the methods section that explains the physiological data supporting its consistency with overall neuronal function. Specifically, in that previous study, neurons with more intense PI staining were less able to maintain normal membrane potentials and generate action potentials. Taken together, we believe adding these new data substantially improve the manuscript while not significantly changing our overall conclusions.

 Regarding why Figure 3C is graphed differently from 3A and 3B, we chose a less cluttered format, given that [% unhealthy cells] in each condition = 100-[% healthy cells]. Plotting both values for each experimental condition seemed redundant. (We plotted both in Figure 2 for illustrative purposes only.) However, if the reviewer insists, we can add [% unhealthy cells] bars to Figure 3C.

# Reviewer report: Reviewer response for version 1

**DOI:** 10.5256/f1000research.137626.r157126

**Published:** 2022-12-23

**Authors:** Malgorzata Beresewicz-Haller

**Affiliations:** 1 Mossakowski Medical Research Institute, Polish Academy of Sciences, Warsaw, Poland

The manuscript from Acharyya and co-workers addresses the very important topic concerning the phenomenon of differential sensitivity of brain regions to excitotoxic stress, as an important component of brain injuries including stroke.

 It appears that, under the influence of the same stimulus, some regions of the brain sustain injury, while others possess internal adaptive mechanisms protecting them against harmful conditions. The former regions, described as vulnerable, include the CA1 region of the hippocampus, layers III and V of the cerebral cortex, and striatal cells and Purkinje cells in the cerebellum; while the latter (resistant) regions include the CA2 and CA3 parts of the hippocampus and the suprachiasmatic nucleus (SCN) mentioned in this manuscript. Although the phenomenon in question has been known for a long time, the mechanisms responsible for it remain unclear, implying a need for further research, all the more so as it is believed that the elucidation of adaptive mechanisms might offer a therapeutic approach to stroke and other neurodegenerative diseases.

 Here, the authors use acute murine brain slices to confirm previous observations regarding the resistance of the SCN to excitotoxic injury. They further show that the susceptibility of the SCN, as well as the hypothalamus and cortex, to injury of this kind proves to be dependent on the time of day. Unlike the authors, who deal with circadian rhythms specifically in the SCN, I was very interested in this result showing that the regions’ susceptibility to excitotoxic injury is greater by night than by day. It is thus unfortunate that this result has received so little exposure, especially since the manuscript gains inclusion in the *Circadian Clocks in Health and Disease* collection.

**My major concerns**

1. A major benefit of the work would be to emphasise that intrinsic resistance to excitotoxic stress is not limited to the SCN, but also involves other regions of the brain. This could lead to possible verification (though only through additional experimentation) of whether the resistance in these different regions is manifested at the same level.
2. Since propidium iodide is not membrane-permeable, it proves useful in differentiating necrotic/apoptotic and healthy cells by reference to membrane integrity. However, it is not useful where differentiation revolves around the degree of damage. I thus have doubts regarding the basis for a distinction to be drawn among PI-positive cells, between those that are damaged or else dead - as was the case for the two image-analysis protocols (IBCC and PACC).
3. Fig. 3 makes clear the rather limited excitotoxic effect of 50 uM NMDA. Thus values in the case of NMDA treatment only differ very slightly from those in the control - to the extent that a question is posed regarding the value of a focus that compares the effectiveness of three different image-analysis protocols, and all the more so since correlations are studied.
4. Comparisons between the SCN and the cortex can prove very misleading. To the best of my knowledge, the cortex has areas within it characterised by vulnerability and resistance to excitotoxic damage, which leaves it crucial for us to have an indication as to which part of the cortex was selected for analysis.

**Minor concerns**

1. Fig. 3C warrants re-examination. The two separate legends here leave this a hard-to-analyse figure. Please also note that the values for the control conditions shown here differ from those noted for control conditions in Fig. 2C - in the face of an obvious expectation that they should be the same. In the current situation, it is difficult to evaluate the results obtained using the mean grey value method, and to relate those to the other two protocols.
2. In regard to Fig. 5, an important complication as differences in sensitivity to excitotoxic damage in the brain regions studied are compared is that, unlike the SCN and hypothalamus, the cortex was not treated with 100 and 500 uM NMDA. The consequence is to limit comparisons to the SCN vs. the hypothalamus, but not the cortex.
3. Here, the effect of excitotoxic stress was analysed 3 hours after the end of NMDA treatment. How was the choice of such a time period justified? Were others also verified? It would be interesting for me to see whether, as the time from the end of NMDA treatment (mainly the 50uM application) extends, the effect of excitotoxic stress between the brain regions studied remains comparable to that observed after 3 h, or is different. In other words, does extending the time from the end of NMDA treatment increase excitotoxic injury?
4. There is confusion over the use of the abbreviation ZT, as once this refers to the incubation time with NMDA, while in another case it is the time before NMDA administration that is meant (when brain slices are perfused with EBSS).
5. The description of the results is sufficiently difficult to follow that readers may well be discouraged. To meet their needs better, I propose that more emphasis be put on the description of the physiological changes taking place, as opposed to the detailing of the presence or absence of statistical significance.

Is the work clearly and accurately presented and does it cite the current literature?

Yes

If applicable, is the statistical analysis and its interpretation appropriate?

Yes

Are all the source data underlying the results available to ensure full reproducibility?

Yes

Is the study design appropriate and is the work technically sound?

Yes

Are the conclusions drawn adequately supported by the results?

Partly

Are sufficient details of methods and analysis provided to allow replication by others?

Yes

Reviewer Expertise:

neuroscience

I confirm that I have read this submission and believe that I have an appropriate level of expertise to confirm that it is of an acceptable scientific standard, however I have significant reservations, as outlined above.

# Response

**Published:** 2023-01-03

**Authors:** Rebecca Prosser

**Affiliations:** University of Tennessee Knoxville, USA

*"Here, the authors use acute murine brain slices to confirm previous observations regarding the resistance of the SCN to excitotoxic injury. They further show that the susceptibility of the SCN, as well as the hypothalamus and cortex, to injury of this kind proves to be dependent on the time of day. Unlike the authors, who deal with circadian rhythms specifically in the SCN, I was very interested in this result showing that the regions’ susceptibility to excitotoxic injury is greater by night than by day. It is thus unfortunate that this result has received so little exposure, especially since the manuscript gains inclusion in the Circadian Clocks in Health and Disease collection."*

**We agree with the reviewer that the circadian rhythms of susceptibility in the cortex in addition to the SCN and AH are extremely interesting and were a primary reason for submitting our manuscript to this collection.  We have added to our existing discussion of these rhythms to further highlight the importance of these findings.**

***"My major concerns"***

1. *"A major benefit of the work would be to emphasise that intrinsic resistance to excitotoxic stress is not limited to the SCN, but also involves other regions of the brain. This could lead to possible verification (though only through additional experimentation) of whether the resistance in these different regions is manifested at the same level."*
**We agree and have added a discussion of other resilient brain regions to the discussion.**
2. *"Since propidium iodide is not membrane-permeable, it proves useful in differentiating necrotic/apoptotic and healthy cells by reference to membrane integrity. However, it is not useful where differentiation revolves around the degree of damage. I thus have doubts regarding the basis for a distinction to be drawn among PI-positive cells, between those that are damaged or else dead - as was the case for the two image-analysis protocols (IBCC and PACC)."*
**We agree and have added references in the discussion section supporting use of PI to assess cell damage and language acknowledge the benefit of using multiple assessment methods in the discussion.**
3. *"Fig. 3 makes clear the rather limited excitotoxic effect of 50 uM NMDA. Thus values in the case of NMDA treatment only differ very slightly from those in the control - to the extent that a question is posed regarding the value of a focus that compares the effectiveness of three different image-analysis protocols, and all the more so since correlations are studied."*
**We agree and have added language to the discussion section to the effect that additional comparisons of the analytical methods going forward would be useful.**
4. *"Comparisons between the SCN and the cortex can prove very misleading. To the best of my knowledge, the cortex has areas within it characterised by vulnerability and resistance to excitotoxic damage, which leaves it crucial for us to have an indication as to which part of the cortex was selected for analysis."*
**We agree and have clarified in the methods section the general cortical areas analyzed and added a brief section in the discussion on other brain regions that have been reported to be resistant to excitotoxic damage.**

***"Minor concerns"***

1. *"Fig. 3C warrants re-examination. The two separate legends here leave this a hard-to-analyse figure. Please also note that the values for the control conditions shown here differ from those noted for control conditions in Fig. 2C - in the face of an obvious expectation that they should be the same. In the current situation, it is difficult to evaluate the results obtained using the mean grey value method, and to relate those to the other two protocols." *
**As also suggested by reviewer #1, we have revised Fig 3 so the legend matches the graph, and clarify in the Fig 3 legend that the Y axis is different from that in Fig 2, although the control data are the same in both. **
2. *"In regard to Fig. 5, an important complication as differences in sensitivity to excitotoxic damage in the brain regions studied are compared is that, unlike the SCN and hypothalamus, the cortex was not treated with 100 and 500 uM NMDA. The consequence is to limit comparisons to the SCN vs. the hypothalamus, but not the cortex."*
**We have added language in the discussion section discussing the lack of treating the cortical tissue with these two concentrations of NMDA and how this affects our comparisons.**
3. *"Here, the effect of excitotoxic stress was analysed 3 hours after the end of NMDA treatment. How was the choice of such a time period justified? Were others also verified? It would be interesting for me to see whether, as the time from the end of NMDA treatment (mainly the 50uM application) extends, the effect of excitotoxic stress between the brain regions studied remains comparable to that observed after 3 h, or is different. In other words, does extending the time from the end of NMDA treatment increase excitotoxic injury?"*
**We agree and have added a discussion of investigating different temporal dynamics in future experiments.**
4. *"There is confusion over the use of the abbreviation ZT, as once this refers to the incubation time with NMDA, while in another case it is the time before NMDA administration that is meant (when brain slices are perfused with EBSS)."*
**We clarify in the methods section that ZT refers to time relative to the animal colony light cycle.**
5. *"The description of the results is sufficiently difficult to follow that readers may well be discouraged. To meet their needs better, I propose that more emphasis be put on the description of the physiological changes taking place, as opposed to the detailing of the presence or absence of statistical significance."*
**We agree and have added descriptive sentences summarizing each set of data alongside the results of the statistical analyses so that the results section is easier to read.**

# Reviewer report: Reviewer response for version 1

**DOI:** 10.5256/f1000research.137626.r155004

**Published:** 2022-12-06

**Authors:** Luz Navarro

**Affiliations:** 1 Neuroendocrinology Laboratory, Department of Physiology, Facultad de Medicina, Universidad Nacional Autónoma de México, Mexico City, Mexico

The manuscript submitted by Acharyya et al. presents interesting and well-substantiated data.  

 The authors compared the excitotoxic resiliency of the suprachiasmatic nucleus, anterior hypothalamus, and cerebral cortex by increasing NMDA concentration in rat brain slices. Also, they analyze this excitotoxic resiliency at different hours of the light-dark cycle. 

 I only have a few minor comments: 

- **Results:** In figure 3C, the colors of the bars do not match those of the legends. It looks like some bars are missing. 
- **Discussion:** Perhaps it could contribute to the discussion; some articles that indicate that the damage caused by an insult to the brain presents diurnal variations in animal models, for example: doi: 10.1007/s12035-017-0524-4; doi: 10.3389/fnins.2020.564992. eCollection 2020; doi: 10.3171/jns.2000.93.1.0082. 

Is the work clearly and accurately presented and does it cite the current literature?

Yes

If applicable, is the statistical analysis and its interpretation appropriate?

Yes

Are all the source data underlying the results available to ensure full reproducibility?

Yes

Is the study design appropriate and is the work technically sound?

Yes

Are the conclusions drawn adequately supported by the results?

Yes

Are sufficient details of methods and analysis provided to allow replication by others?

Yes

Reviewer Expertise:

Neuroprotection, diurnal rhythms

I confirm that I have read this submission and believe that I have an appropriate level of expertise to confirm that it is of an acceptable scientific standard.

## References

[1] : Time-of-Day Dependent Neuronal Injury After Ischemic Stroke: Implication of Circadian Clock Transcriptional Factor Bmal1 and Survival Kinase AKT. *Mol Neurobiol* .2018;55(3) : 10.1007/s12035-017-0524-4 2565-2576 10.1007/s12035-017-0524-4 28421530

[2] : Diurnal Variation Induces Neurobehavioral and Neuropathological Differences in a Rat Model of Traumatic Brain Injury. *Frontiers in Neuroscience* .2020;14: 10.3389/fnins.2020.564992 10.3389/fnins.2020.564992 33132827 PMC7550533

[3] : Temporal changes in sensitivity of rats to cerebral ischemic insult. *J Neurosurg* .2000;93(1) : 10.3171/jns.2000.93.1.0082 82-9 10.3171/jns.2000.93.1.0082 10883909

[4] : Diurnal variation of NMDA receptor expression in the rat cerebral cortex is associated with traumatic brain injury damage. *BMC Res Notes* .2018;11(1) : 10.1186/s13104-018-3258-0 150 10.1186/s13104-018-3258-0 29467028 PMC5822486

# Response

**Published:** 2023-01-03

**Authors:** Rebecca Prosser

**Affiliations:** University of Tennessee Knoxville, USA

- ***"Results:** In figure 3C, the colors of the bars do not match those of the legends. It looks like some bars are missing." *
  We apologize for the oversight and have revised the figure so the legend matches the bars.
- **" *Discussion:*** *Perhaps it could contribute to the discussion; some articles that indicate that the damage caused by an insult to the brain presents diurnal variations in animal models, for example: doi: 10.1007/s12035-017-0524-4; doi: 10.3389/fnins.2020.564992. eCollection 2020; doi: 10.3171/jns.2000.93.1.0082.*" 
  We agree and have included this in the discussion.
